# Smart Nanomaterials for Treatment of Biofilm in Orthopedic Implants

**DOI:** 10.3389/fbioe.2021.694635

**Published:** 2021-09-13

**Authors:** Qimin Hong, Shicheng Huo, Haozheng Tang, Xinhua Qu, Bing Yue

**Affiliations:** Department of Bone and Joint Surgery, Department of Orthopaedics, Renji Hospital, School of Medicine, Shanghai Jiao Tong University, Shanghai, China

**Keywords:** nanomaterials, stimuli-responsive, biofilm, nanomedicine, infection

## Abstract

Biofilms refer to complex bacterial communities that are attached to the surface of animate or inanimate objects, which highly resist the antibiotics or the host immune defense mechanisms. Pathogenic biofilms in medicine are general, chronic, and even costly, especially on medical devices and orthopedic implants. Bacteria within biofilms are the cause of many persistent infections, which are almost impossible to eradicate. Though some progress has been made in comprehending the mechanisms of biofilm formation and persistence, novel alternative compounds or strategies and effective anti-biofilm antibiotics are still lacking. Smart materials of nano size which are able to respond to an external stimulus or internal environment have a great range of applications in clinic. Recently, smart nanomaterials with or without carriage of antibiotics, targeting specific bacteria and biofilm under some stimuli, have shown great potential for pathogenic biofilm and resident bacteria eradication. First, this review briefly summarizes and describes the significance of biofilms and the process of biofilm formation. Then, we focus on some of the latest research studies involving biofilm elimination, which probably could be applied in orthopedic implants. Finally, some outstanding challenges and limitations that need to be settled urgently in order to make smart nanomaterials effectively target and treat implant biofilms are also discussed. It is hoped that there will be more novel anti-biofilm strategies for biofilm infection in the prospective future.

## Introduction

Biofilm formation, an ancient and indispensable feature of microorganisms, represents a protected mode of growth (Hall-Stoodley et al., 2004). Since 1982, the first time when the involvement of biofilms in medical devices (cardiac pacemakers) was observed by electron microscopy, almost all types of indwelling devices have been related to the occurrence of bacterial or fungal biofilms (Hall-Stoodley et al., 2004; Lebeaux et al., 2014). It is now estimated that 60–80% of bacterial infections in humans are caused by bacterial biofilms (Childs, 2008). The morbidity and mortality chronically associated with biofilm infection are very high (Drancourt et al., 1993), and annually, the treatment cost of biofilm infection is more than $6 billion (Ehrlich et al., 2005).

Approximately, 1 million orthopedic prosthetic devices are implanted within the United States each year, and 2% of these were infected (Ehrlich et al., 2005; Matthew et al., 2018). Periprosthetic joint infection (PJI) is a serious complication for orthopedic implant-related operations and thought to be largely due to biofilms that can grow on the surfaces of the implant (Gbejuade et al., 2015; Tzeng et al., 2015), with an estimation that 80% of all bacterial PJIs involve biofilm formation (Shoji and Chen, 2020). *Staphylococcus aureus*, coagulase-negative *Staphylococcus*, *Streptococcus*, and *Pseudomonas* are the common pathogens among these biofilm infections (Schinsky et al., 2008; Tzeng et al., 2015). Despite the high incidence of reinfection (Shoji and Chen, 2020), in many cases, the only efficient solution is the removal of the implanted device or surgically excising the infected tissue (Nistico et al., 2014), which is not only extremely long and painful for patients but also a considerable financial burden for the health-care system (Nistico et al., 2014). In addition to causing orthopedic implant–associated infections, biofilms are also highly related to urinary tract infections (UTI) (Flores-Mireles et al., 2015), of which the biggest risk factor is the indwelling catheter (Shuman and Chenoweth, 2018). Given that many patients with joint replacement require catheterization during surgery, knowing how to prevent urinary tract infections is crucial (Khan et al., 2020b).

Nanoparticles are a highly promising approach to biofilm therapy (Kumar et al., 2018; Eleraky et al., 2020), with outstanding capacity to directly kill bacteria or release bactericides by well adjusting their chemical composition, size, and surface charge (Weir et al., 2008; Khan et al., 2020a; Khan et al., 2020c; Khan et al., 2021). Some smart nanomaterials that respond to unique microenvironments of the harsh biofilm matrix or artificial stimulus can provide unparalleled flexibility to carry, retain, and release bactericides exactly when and where needed most (Benoit et al., 2019). Here, we summarize and describe the significance of biofilms as well as the process of biofilm formation. Then we will focus on some of the latest research on smart nanomaterials killing bacteria and eliminating biofilms, which probably could be applied in orthopedic implants, in the further paragraphs. Finally, outstanding challenges and limitations that need to be settled urgently are also discussed.

## Significance of Biofilms

Biofilms can be regarded as layered aggregates of microbial cells and cellular products (Costerton et al., 1978; Rodríguez et al., 2011) with a three-dimensional polymer (Koo et al., 2017) network clinging to solid surfaces (Kumar et al., 2019) (surfaces of implants), which can provide important structural support and protection for microbial communities (Flemming et al., 2007; Flemming and Wingender, 2010) and an environment for the exchange of genetic material between microbial individuals (Rodríguez et al., 2011). The established biofilm architecture consists of microbial cells and an extracellular polymeric substance (EPS) matrix, of which the EPS accounts for 90% of the mass with the rest being microbial cells (Flemming and Wingender, 2010). It is worth noting that water, bound in capsules of microbial cells or existing as a solvent with physical properties (Schmitt and Flemming, 1999), makes up a large proportion of the biofilm matrix (up to 97%) (Zhang et al., 1998; Sutherland, 2001). Although the physical and chemical compositions of the EPS of different microorganisms vary, except for water, biofilm substrates generally include all major macromolecules, such as exopolysaccharides, proteins, extracellular DNA (eDNA), lipids, and phospholipids (Toyofuku et al., 2012; Zoubos et al., 2012; Tzeng et al., 2015). In fact, absorbed nutrients, metabolites of microorganisms, products of cell lysis, and particulate matter and debris from the surrounding environment may also be present (Zoubos et al., 2012).

Microbial cells within a biofilm can resist most adverse environments (Le Magrex-Debar et al., 2000; Espeland and Wetzel, 2001; Teitzel and Parsek, 2003; Karol and Hamilton, 2010) (e.g., UV light exposure, heavy metal toxicity, acidity, dehydration, and salinity changes) and host immune defenses (Leid et al., 2002; Wagner et al., 2003) (e.g., opsonization, phagocytosis, and complement-mediated lysis). In addition, biofilm bacteria also display a characteristic ability to withstand antibiotics (Walsh, 2000; Stewart and William Costerton, 2001) compared to planktonic ones (Nickel et al., 1985; Lewis, 2001; 2008) because of 1) mechanical and physicochemical properties of the biofilm matrix reducing or delaying antibiotic diffusion (Mugabe et al., 2006; Halwani et al., 2007; Høiby et al., 2010; Li et al., 2013) and 2) the depletion of nutrients and/or oxygen as well as accumulation of waste product causing bacteria to enter a stationary state, which is insensitive to antimicrobial agents (Zoubos et al., 2012). Biofilms can release extracellular molecules to change gene expression of virulence factors through quorum sensing (Shoji and Chen, 2020). Additionally, bacteria in biofilms can increase mutation frequency to avoid host defenses (Driffield et al., 2008), increase *β*-lactamase activity (Ciofu, 2003), increase efflux pump activity (Pamp et al., 2008), and exchange plasmids for transfer of genes for antibiotic resistance and virulence factors. The remaining biofilm matrix scaffold where microbial cells have been inactivated may facilitate subsequent colonization of other microorganisms or serve as a source of nutrients (Koo et al., 2017; Arciola et al., 2018). All of these contribute to a significant number of therapeutic difficulties encountered in clinical settings.

## Formation Process of Biofilm

**FIGURE 1:**
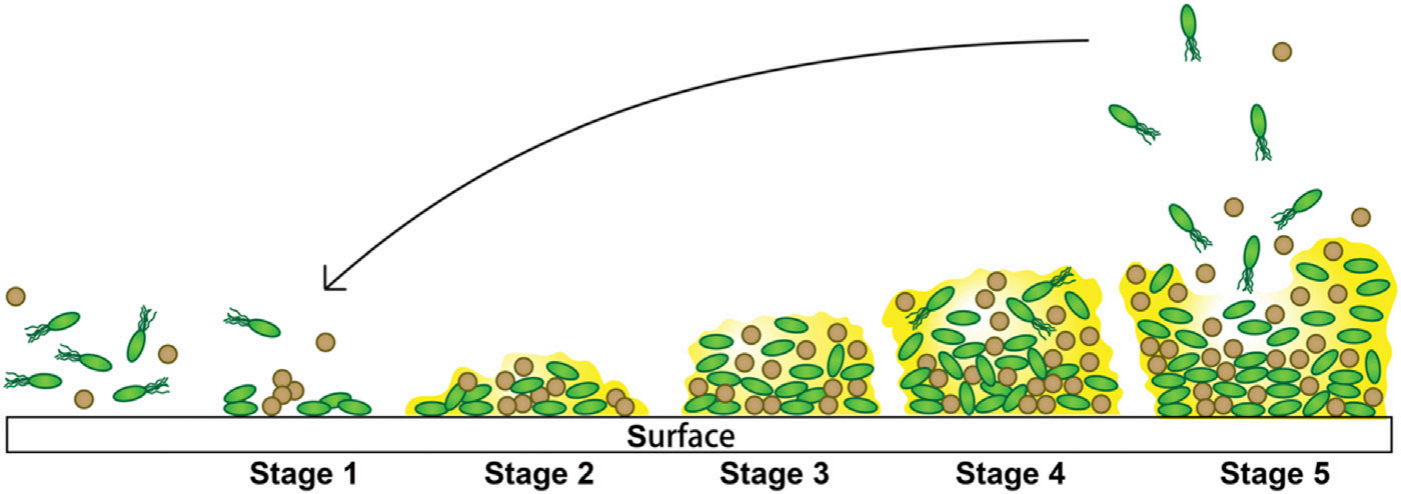
Steps involved in biofilm formation. 1) Initial attachment. 2) Extracellular polymeric substance production. 3) Biofilm development. 4) Biofilm maturation. 5) Dispersion from the biofilms.

In implant-associated infections, the implant will trigger a local tissue response and generate a niche of immune depression, which predisposes Table 1 the implant to microbial colonization (Menkin, 1931; Anderson, 2016). The formation of biofilm on the surface is a dynamic stepwise process, and roughly, it can be divided into five stages (Figure 1) (Genevaux et al., 1996; O’Toole and Kolter, 1998; Pratt and Kolter, 1998; Watnick and Kolter, 1999; Hall-Stoodley et al., 2004; Costerton, 2005; Zoubos et al., 2012; Koo et al., 2017; Arciola et al., 2018) as follows:

**TABLE 1:** Stimuli-responsive nanomaterials with antibacterial properties.

Nanosystem	Stimuli type (s)	Main composition (s)	Bacterial strains	Antibacterial mechanism	Antibacterial effect	Development	Reference
Magnetic iron oxide nanoparticles (MNPs)	Magnetism-responsive	Fe_3_O_4_	MRSA	Mechanical disruption; magnetic hyperthermia	5 log_10_ reduction in biofilm bacteria	*in vitro*	Li et al. (2019a)
Superparamagnetic iron oxide nanoparticles (SPIONs)	magnetism-responsive	BioMag^®^ Superparamagnetic Iron Oxide	*P. aeruginosa*	Magnetic hyperthermia	More than 4 log inactivation of the PA01 biofilm	*in vitro*	Park et al. (2011)
Magneto-responsive gallium-based liquid metal (LM) droplets	Magnetism-responsive	68.5 wt% gallium; 21.5 wt% indium; and 10 wt% tin	*S. aureus*; *P. aeruginosa*	Mechanical disruption	Inactivating 99% of both species of bacteria; disintegrating biofilms	*in vitro*	Elbourne et al. (2020)
Magnetic nanocomposites	Magnetism-responsive	Selenium; iron oxide; and chitosan	*S. aureus*	Mechanical disruption; ROS; and thiol depletion	Relative ratio of dead-to-live bacteria in nanocomposites was 400.0%	*in vitro*	Li et al. (2020b)
Silver ring–coated super paramagnetic iron oxide NPs	Magnetism-responsive	Iron oxide nanoparticles; silver nanoparticles	*S. aureus; S. epidermidis*	Mechanical disruption; Ag^+^	Enhancing the antimicrobial activities of Ag	*in vitro*	Mahmoudi and Serpooshan, (2012)
Magnetite hybrid nanocomplexes	Magnetism-responsive	Iron oxides; Ag	*E. coli; P. aeruginosa*	Mechanical disruption; Ag^+^	A significant reduction of the biofilm and viable bacterial cells	*in vitro*	Zhang et al. (2019)
PEL1-CS-Fe3O4	Magnetism-responsive	Fe_3_O_4_; phage PEL1; and magnetic colloidal nanoparticle clusters	*E. coli; P. aeruginosa*	Mechanical disruption; biological inhibition	Modest killing of the bacteria (≈40%); remarkable reduction in biofilm (88.7%)	*in vitro*	Li et al. (2017)
Antimicrobial magnetic thermotherapy platform	Magnetism-responsive	Fe_3_O_4_; anti-protein A antibody	*S. aureus*	Magnetic hyperthermia; biological inhibition	99% killing efficiency *in vitro*; a significant reduction of the *S. aureus in vivo*	*in vitro*; *in vivo*	Kim et al. (2013)
Deoxyribonuclease-decorated gold nanoclusters	Light-responsive	DNase; Au	*S. aureus; P. aeruginosa*	Biological inhibition; photothermal therapy; and photodynamic therapy	Removing 80% biofilms; killing ∼90% shielded bacteria	*in vitro*	Xie et al. (2020b)
Protease-conjugated GNRs (PGs)	Light-responsive	Bromelain; Au	*E. coli*; *S. aureus*	Photothermal therapy; biological inhibition	Decreasing bacterial growth (96.8% for *E. coli*, 97.9% for *S. aureus*); removing 70.5% (*E. coli*); and 93.3% (*S. aureus*) of biofilm mass	*in vitro*	Li et al. (2019b)
RP–IR780–arginine–glycine–aspartic acid–cysteine coating	Light-responsive	Red phosphorus; IR780	*S. aureus*	Photothermal therapy; photodynamic therapy	96.2% antibacterial efficiency *in vivo*	*in vitro*; *in vivo*	Tan et al. (2018)
TiO2 nanorod arrays	light-responsive	TiO_2_	*S. aureus*; *E. coli*	Photothermal therapy; photodynamic therapy; and physical destruction	About 100 and 99.9% against *E. coli* and *S. aureus in vitro*; excellent antibacterial activity *in vivo*; and eradication of biofilms	*in vitro*; *in vivo*	Zhang et al. (2021)
Titanium-containing composite material surface	Light- and thermo- responsive	VCL-co-QAS-co-PEGMA-co-VTMO; QAS	*S. aureus; E. coli*	Physical and chemical effects; QAS	Outstanding antibacterial properties (98% for both *S. aureus* and *E. coli*); anti-adhesive property (99.86% for *S. aureus* and 97.08% for *E. coli*)	*in vitro*; *in vivo*	Lin et al. (2021)
Multifunctional catechin@ZIF-L nanocomposite (CA@ZIF-L)	pH-responsive	Catechins; Zn; ZIF	MRSA	Catechins; Zn^2+^	Eradicating biofilms in a dose-dependent manner	*in vitro*	Raju et al. (2020)
Surface-adaptive, antimicrobially loaded, micellar nanocarriers	pH- and enzyme-responsive	Triclosan; poly (ethylene glycol) (PEG); and β-amino ester	*S. aureus*	Triclosan	More effective in killing staphylococci deep into a biofilm	*in vitro*	Liu et al. (2016)
PPD@CDLys	pH-responsive	2,3-dimethylmaleic anhydride (PPD); calcined l-lysine powder (CD_Lys_)	*S. aureus*; *E. coli*	PPD; ROS	Effectively disrupting the mature biofilm; inactivating the embedded bacteria in a short time	*in vitro*	Li et al. (2020a)
Surface charge switchable supramolecular nanocarriers (*α*-CD-Ce6-NO-DA)	pH-responsive	*α*-Cyclodextrin (*α*-CD); nitric oxide prodrug; chlorin e6 prodrug; and poly (ethylene glycol) (PEG)	MRSA	NO; ROS; RNS; and photodynamic therapy	The bactericidal rate of the biofilm was 99.92 ± 0.72% *in vitro*; having much better bactericidal effect *in vivo*	*in vitro*; *in vivo*	Hu et al. (2020)
Surface-adaptive mixed charged gold nanoparticle (AuNP-N-C)	pH- and light-responsive	Au; (10-mercaptodecyl) trimethylammonium bromide; and 11-mercaptoundecanoic acid	MRSA	Photothermal therapy	Effectively adhering to bacteria; rapidly aggregating in MRSA biofilm, exhibiting great bactericidal effects	*in vitro*; *in vivo*	Hu et al. (2017)
LBL@MSN-Ag nanocoating	Enzyme-responsive	Ag; MSN; poly-l-glutamic acid; and polyallylamine hydrochloride	*S. aureus*	Ag^+^	Having superior antibacterial capacity (>95%) *in vitro*; modified Ti implants, and effectively treated infections *in vivo*	*in vitro*; *in vivo*	Ding et al. (2020)
Size/surface charge-adaptive micelles	pH- and enzyme-responsive	Cationic copolymers; azithromycin; and *cis*-aconityl-d-tyrosine	*P. aeruginosa*	d-tyrosine; azithromycin	Micelles disrupt biofilms (85% dispersal percentage *in vitro*) and eliminate bacteria	*in vitro*; *in vivo*	Chen et al. (2019)
Enzyme-responsive copolymer micelles	Enzyme-/toxin-responsive	Poly vinyl caprolactam; polyethylene glycol; chlorhexidine	*S. aureus*; MRSA; *S. epidermidis*	Chlorhexidine	Reducing biofilm biomass (>60%)	*in vitro*	Albayaty et al. (2019)
Gold nanoparticle–stabilized liposomes	Toxin-responsive	Au; chitosan; liposomes; and vancomycin	MRSA	Vancomycin	Inhibiting MRSA growth as effectively as an equal amount of free vancomycin	*in vitro*	Pornpattananangkul et al. (2011)

Stage 1: Initial attachment to the surface. The association with the surface of this stage is loose, transient, and reversible and only takes a few seconds, which occurs by various mechanisms including Brownian motion, chemoattraction, and weak van der Waals forces and facilitated by force-producing organelles such as type IV pili and flagella. Besides, a variety of environmental signals (surface composition and roughness, hydrodynamics, hydrophobicity, temperature, osmolarity, nutrients, pH, oxygen concentration, and irons) probably influence this initial interaction. In this stage, the bacterial cells show a logarithmic rate of growth.

Stage 2: Extracellular polymeric substance production. Bacteria begin to multiply when they successfully adhere to the surface. During this stage, bacteria are able to “communicate” with other individuals and activate the genetic mechanism of EPS production through intercellular signals sent by them. Then, the EPS accumulates and progressively generates multiple layers, during which these aggregates can capture nutrients and planktonic bacteria, resulting in robust adhesion. The production of the extracellular polymeric substance is a necessary condition for the stabilization of biofilms.

Stage 3: Biofilm architecture development, also known as maturation I. The bacteria gradually gather into small colonies in this stage, in which the cell-to-cell connection becomes closer and more complicated.

Stage 4: Biofilm architecture maturation, also known as maturation II. The biofilm of this stage will generally become larger to reach its thickness limit, until it gets to the next stage.

Stage 5: Dispersion from the biofilms. When biofilm environmental conditions are unfavorable, this stage begins. Some of the biofilm bacteria with transient motility develop planktonic phenotypes and disperse away from the biofilm to find a surface to recolonize, where the conditions are more favorable. There are three common strategies for bacteria to disperse: “swarming/seeding dispersal,” in which individual cells are released from biofilms into the surrounding substratum or the fluid; “clumping dispersal,” in which aggregates of cells are shed as clumps or emboli; and “surface dispersal,” in which biofilm structures move across surfaces.

## Nanoparticle-Based Biofilm Treatments

Many biofilm management strategies currently being devised in the clinic and used by surgeons are largely based on an approach from cancer treatment: early and aggressive physical removal and conventional antibiotic-based therapy or topical delivery of high and sustained antimicrobials (Høiby et al., 2015). Considering the difficulty in the early diagnosis of biofilm infection, poor biofilm matrix penetration of conventional applied drugs, altered microenvironment of biofilm influencing the antimicrobials activity, and the rapid development of bacterial resistance, preventing or treating pathogenic biofilms is challenging. Despite extensive efforts in research and enormous investment of resources, new classes of antibiotic development have been slow. Current advances in chemical engineering and nanotechnology offer nanomaterials a promising prospect to combat bacteria (Koo et al., 2017; Wang et al., 2017; Baptista et al., 2018), by controlling the composition, size, shapes, structure, surface area, and chemistry to have antimicrobial ability. In addition to their multiple antibacterial activity mechanisms (Baptista et al., 2018), such as 1) direct interaction with the bacterial cell wall; 2) generation of reactive oxygen species (ROS); 3) triggering of innate and adaptive host immune responses; and 4) induction of intracellular effects, nanomaterials with high surface-to-volume ratios and multivalent interactions can also act as carriers for antibiotics or assist in delivering novel drugs (Baptista et al., 2018). Moreover, numerous pieces of experimental evidence show that nanoparticles are capable of disrupting bacterial membranes and can hinder biofilm formation, thus reducing the survival of the microorganism (Baptista et al., 2018).

In general, nanomaterials used for antimicrobial applications can be classified into metal and metallic oxide nanoparticles, carbon-based nanomaterials, polymeric nanoparticles, nanocomposites, nano emulsions, lipid nanoparticles, and smart nanomaterials (Qayyum and Khan, 2016; Joshi et al., 2020; Makabenta et al., 2021). Smart nanomaterials, with the ability to change their characteristics that allow them to exert antimicrobial action or control drug release under the condition of endogenous stimuli or external stimuli (e.g., pH, bacterial toxins, redox potential, enzymatic activation, magnetic fields, light, temperature, and ultrasound), have been rapidly developed over the years (Karimi et al., 2016; Makabenta et al., 2021).

### Magnetic Responsive Nanomaterials

Magnetic fields can penetrate body tissues, so they are commonly used in MRI for body imaging; in addition to that, external magnetic stimulation can also control the magnetic responsive nanomaterials for treating biofilm infection (Yang et al., 2018). There are two strategies: magnetic field–induced hyperthermia for drug release (Karthikeyan et al., 2018) and magnetic field–guided drug targeting (Schleich et al., 2015), the basic mechanisms of which involve their ability to generate heat via alternating magnetic frequency.

Exposure of magnetic iron oxide nanoparticles to magnetic field will cause a localized rise in temperature which destroys biofilms through static friction, dispersing embedded bacterial cells (Li et al., 2019a). Park et al. (2011) have demonstrated superparamagnetic nanoparticles that have anti-biofilm properties, which are able to reduce the viable *P. aeruginosa* cells within a thick biofilm more than a four log under a magnetic field (3 kA m^−1^, 493 kHz) for 8 min. Elbourne et al. (2020) utilized magneto-responsive gallium-based liquid metal droplets, known as “Galinstan.” When exposed to a low-intensity rotating magnetic field, the magnetic droplets transform from spheres to high–aspect ratio rods and star-like particles with nanoscale sharp edges to effectively remove the majority of biofilms formed by *S. aureus*, and *P. aeruginosa* bacteria and meanwhile pierce the bacterial cell wall. Similarly, in Li’s work (Li et al., 2020b), a new magnetic nanocomposite was synthesized, which combines selenium nanoparticles (SENPs) with iron oxide nanoparticles (IONPs). Depending on the pH of the reaction mixture, the selenium nanoparticles can be nearly spherical or rod-shaped. Nanocomposites showed excellent anti-biofilm property in the presence of external magnetic field toward *S. aureus*, and the toxicity to human cells was significantly low. The results show that the relative ratio of dead-to-live bacteria in nanocomposites (400.0%) was much higher than that of SENPs (51.6%) and IONPs (60.0%).

Other studies have been able to successfully conjugate antibiotics onto magnetic nanomaterials for pathogen treatment. Mahmoudi and coworkers developed silver ring–coated superparamagnetic iron oxide NPs (SPIONs) which under an external magnetic field diffuse into deep biofilm matrix and exhibit improved activity against biofilm infection, demonstrating high antimicrobial ability without causing damage to the healthy cells (Mahmoudi and Serpooshan, 2012). Similar to the previous study, Zhang et al. (2019) synthesized a kind of nanocomposite, with Fe_3_O_4_ as an outer shell surrounded by an inner core of nanosilver as the antimicrobial agent. In this case, nanocomposites can be magnetically activated for biofilm penetration, and they demonstrated a significant reduction in the biofilm and viable bacterial cells in a test against biofilms composed of *E. coli* and *P. aeruginosa.* Furthermore, Li et al. (2017) utilized chitosan-coated Fe_3_O_4_ colloidal nanoparticle clusters (CS-Fe_3_O_4_), conjugated with the polyvalent bacteriophage PEL1, to penetrate biofilms of *P. aeruginosa* and *E. coli*. The PEL1 were allowed to kill the bacterial cells within the biofilm, with the help of CS-Fe_3_O_4_ nanoclusters physically disrupting and penetrating the biofilm. Through modest killing of the bacteria (≈40%), they achieved a remarkable reduction in biofilm coverages, by 88.7 ± 2.8%. Kim et al. (2013) even conjugated iron oxide nanoparticles with anti-protein A antibody for the targeted treatment of *S. aureus* biofilms. They demonstrated positive results, with a killing efficiency at above 99% following an alternating magnetic field of 31 and 40 kA m^−1^ thal dose *in vitro* and a significant reduction of *S. aureus in vivo* of the mouse model.

### Light-Responsive Nanomaterials

Several wavelengths of light [e.g., ultraviolet (Brown et al., 2009), visible (Hossion et al., 2013), and near-infrared light (Liu et al., 2017)] are used against bacteria (Raza et al., 2019; Pham et al., 2020). Owing to better penetration ability and limited damage to cells, NIR is of more potential benefit than the others (Xiang et al., 2018), which involves three different mechanisms: the photothermal effect, two-photon absorption, and upconverting nanoparticles (Raza et al., 2019; Pham et al., 2020). The photothermal agent can transfer light to heat through the photothermal effect and then stimulate the heat-sensitive material to break nanostructures, resulting in drug release at the bacterial infection site or other response; meanwhile, the produced heat and reactive oxygen species (ROS) can be effective against pathogenic bacteria (Li et al., 2018).

Xie et al. (2020b) demonstrated that deoxyribonuclease (DNase)-decorated gold nanoclusters (DNase–AuNCs) could disperse biofilms and kill encapsulated bacteria. DNase degrades the bacterial extracellular matrix to inhibit biofilm formation or destroy the formed biofilm matrix, allowing the AuNCs to access the encapsulated bacteria to perform combined phototherapy under the excitation of 808-nm lasers. Moreover, fluorescent DNase–AuNCs can be used to trace or detect bacteria, thanks to their interaction with the pathogens.

Li et al. (2019b) reported a promising antimicrobial smart nanomaterial that integrates the properties of protease (bromelain) with a gold nanorod scaffold, named protease-conjugated GNRs (PGs). In addition to achieving thermal degradation and elimination of biofilms as well as exotoxins, PGs also improve the activity of a conjugated mesophilic protease, by employing hyperthermia generated by conversion of NIR. It exhibited a broad spectrum of antibacterial activity against both *E. coli* and *S. aureus*.

Tan et al. (2018) prepared the fibrous red phosphorus (RP) film, a red phosphorus–IR780–arginine–glycine–aspartic acid–cysteine (RGDC) coating, on the titanium bone implant’s surface through chemical vapor deposition. Red phosphorus has great ability of biocompatibility, while IR780 as an NIR photosensitizer can produce singlet oxygen enhancing the temperature sensitivity of *S. aureus* biofilm. This approach eradicated the biofilm through near-infrared (808 nm) photothermal therapy both *in vitro* and *in vivo* without damaging the normal tissue as well as reached an antibacterial efficiency of 96.2% *in vivo.* Meanwhile, RGDC even improved the cell adhesion, proliferation, and osteogenic differentiation.

TiO_2_ nanorod arrays with high photothermal conversion ability can produce a small amount of ROS, which is antibacterial. In the work of Zhang et al. (2021), TiO_2_ nanorod arrays combined with the irradiation of 808 near-infrared (NIR) light demonstrated the ability to eradicate single-species biofilms through a combination of photothermal therapy, photodynamic therapy, and physical destruction to bacteria. Physiologically, with only 15 min of irradiation, the combination of high temperature, ROS, and nanorod puncture produced excellent antimicrobial properties toward *S. aureus* or *E. coli* on titanium, which they demonstrated *in vitro* and *in vivo* experiments. At the same time, the nanorod arrays improved cell adhesion, proliferation, and osteogenic differentiation, thereby accelerating bone regeneration.

Photo response nanomaterials also have the ability to reduce bacterial adhesion. Lin et al. (2021) synthesized a light-responsive nanocomposite, by grafting thermo-responsive P [vinylcaprolactam (VCL)–co-polyethylene glycol methacrylate (PEGMA)–co-alkyl-dimethyl tertiary amine (QAS)–co-vinyltrimethoxysilane (VTMO)] copolymer on TiO_2_ nanotubes/titanium (TNTs)/Ti surface (VCL–co-PEGMA–co-QAS–co-VTMO). The thermal response resulted in conformational changes in polymer molecules under water, while the light response resulted in the formation of ROS on the surface of the composites, both of which reduced bacterial adhesion (99.86% for *S. aureus* and 97.08% for *E. coli*, respectively). Due to the combination of antimicrobial QAS, the composite surface showed significant antimicrobial activity of 98%, both against *S. aureus* and *E. coli*.

### pH-Responsive Nanomaterials

pH values vary in many specific, physiological or pathological state. pH levels diversify in various segments of our body; pH of saliva ranges 6.5–7.5, and the pH changes from 4–6.5 (stomach) to 5–8 (intestine) along the gastrointestinal tract (Date et al., 2016; Aflori, 2021). Additionally, for example, bacterial infections present with an acidic pH in the range 6.0–6.6, an inflamed tissue has a pH value of 6–7, and pH values are 7.4–5.4 in chronic wounds (Xie et al., 2020a; Aflori, 2021). Smart nanomaterials responding to diverse pH show great functional properties and have important application value in the biomedical field. For instance, pH-responsive nanocarriers were used to deliver hydrophobic drugs to the biofilm matrix, which consisted of a cationic outer shell to bind with the EPS and a pH-responsive hydrophobic inner shell to release encapsulated farnesol molecules on demand (Horev et al., 2015). A 2-fold increase in the treatment efficacy of biofilms was reached by these nanomaterials, compared to the drug use alone.

In a study of Raju et al. (2020), catechins were loaded onto zeolitic imidazole frameworks (ZIFs) to synthesize pH-responsive nanocarriers. Acidic pH within the biofilm favors the disintegration of ZIF-L framework, leading to the release of catechin and Zn^2+^ ions. Catechin can destabilize the biofilm matrix through damage to the membrane or weaken the biofilm formation by suppressing quorum sensing and enzyme glucosyl transferase. In addition, Zn^2+^ also exhibited anti-biofilm activity by impairing the swarming ability and exopolysaccharide production, and the synergistic effect of anti-biofilm with catechin. Liu et al. (2016) developed surface-adaptive, pH-responsive, and mixed-shell polymeric micelles as nanocarriers for hydrophobic antimicrobials (Triclosan). These nanocarriers can penetrate *S. aureus* biofilms at physiological pH, adapt a positive charge under acidic pH conditions, and target themselves to negatively charged bacterial cell surfaces where they are hydrolyzed by bacterial lipases to release the encapsulated drug, bypassing biofilm recalcitrance to antimicrobial penetration. In a work of Li et al. (2020a), they designed a pH-sensitive anti-biofilm nanosystem based on carbon dots (PPD@CDLys), self-assembled by a negatively charged shell [poly (ethylene glycol)-COOH-polyethylenimine-2,3-dimethylmaleic anhydride (PPD)] and a positively charged core [amines on the surface of carbon dots derived from the ashes of calcined l-lysine powder (CD_Lys_)]. The outer copolymer reversed to be positively charged by amide hydrolysis in a mildly acidic environment, making PPD@CD_Lys_ permeate into the dense biofilm. Under the stimulation of the acidic microenvironment of the biofilm, PPD@CD_Lys_ disintegrated, protonized the -NH_2_-ended shell polymer, and transformed into a cationic antibacterial agent. Meanwhile, the released CDs can also produce ROS to decompress the EPS. Under the synergistic antibacterial effects of cation and ROS, the formation of *S. aureus* biofilm can be effectively inhibited and the mature biofilm can be destroyed quickly.

The surface charge switchable nanocarriers can exhibit outstanding synergistic photodynamic eradication of the MRSA biofilm. In research of Hu et al. (2020), they reported a supramolecular nanocarrier (*α*-CD-Ce6-NO-DA), integrating the *α*-cyclodextrin-conjugated NO prodrug and Ce6 prodrug into PEG block polypeptide copolymer, to target the biofilm microenvironment. At acidic biofilm pH (5.5), *α*-CD-Ce6-NO-DA nanocarriers become positively charged (at physiological pH of 7.4, have negatively charged surfaces), facilitating effective penetration into the biofilm and adhesion to the negatively charged bacterial surfaces, and then release NO molecules. NO could dramatically reduce the concentration of GSH in biofilm to enhance the PDT efficiency. Moreover, light-triggered ROS reacted with NO, producing the RNS (ONOO−) with stronger bactericidal ability, which further improves the PDT efficiency. In a similar study, Hu et al. (2017) also designed a kind of surface-adaptive mixed charged zwitterionic gold nanoparticle (abbreviated as AuNP-N-C) to investigate the effect of an acidic trigger approach onto the effective adherence and enhanced photothermal ablation of MRSA biofilm, though bare damage to surrounding healthy tissues.

### Enzyme/Toxin-Responsive Nanomaterials

Among all stimuli-responsive systems, enzyme-responsive systems are suitable for biomedical applications owing to their high selectivity and specificity (Aflori, 2021). The sites of bacterial infection are often full of enzymes or toxins that block the penetration of antibiotics and inactivate them (Aflori, 2021). Notably, many antimicrobial research studies have employed enzyme-responsive nanocarriers (Aflori, 2021), which provide a hydrophilic environment to stabilize the hydrophobic antimicrobials and release the encapsulated drug when the nanocarrier matrix was enzymatically degraded in biofilms.

For instance, Ding et al. (2020) reported a kind of a titanium-based implant containing mesoporous silica nanoparticles loaded with silver nanoparticles coated with multilayer layers of poly (l-glutamic acid) (PG) and polyallylamine hydrochloride, an enzyme-sensitive nanomaterial designed to treat infections associated with *S. aureus* and to facilitate the growth of bone tissue *in vivo*. Chen et al. (2019) designed pH- and lipase-sensitive hybrid micelles, which grafted d-tyrosine and azithromycin. The anionic surface of micelles can reduce nonspecific interactions with blood proteins and cells, which prolongs blood circulation and enhances the accumulation at the infection area. In response to bacterial lipases and the acidic pH environment of *P. aeruginosa* biofilms, micelles shrank in size, reversed charge, and d-tyrosine released to disperse the dense biofilm matrix, along with azithromycin releasing to destruct bacterial cells. In addition, Albayaty et al. (2019) designed a kind of copolymer micelle as a nanocarrier that is susceptible to lipases/esterases produced by bacteria, such as *S. aureus* and *P. aeruginosa*, successfully achieving targeted release of chlorhexidine (CHX) in bacterial biofilms. This method not only further increased permeability of CHX (71%) but also promoted maximum reduction in biofilm biomass (>60%).

In addition to enzyme-responsive nanoplatform, nanomaterials can be designed to trigger antibiotic release of antibiotics after exposure to bacterial toxins. For example, Pornpattananangkul et al. (2011) fabricated bacterial toxin–responsive AuNP-stabilized phospholipid liposomes (AuChi liposomes). Chitosan-functionalized AuNPs were adsorbed on the liposomal surfaces to provide stability and prevent undesirable antibiotic leakage. In the presence of *α*-toxin secreted by *S. aureus*, AuChi liposomes released vancomycin that effectively inhibited their growth.

## Immunoregulation Effect of Nanomaterials

Macrophages are immune cells of plasticity and heterogeneity that are essential regulators of the host defense in humans (Taylor et al., 2005). Macrophages in the resting state (M0) can respond to various local microenvironments and differentiate into two kinds of activated states of macrophages: pro-inflammatory (M1) or anti-inflammatory (M2), which is defined as macrophage polarization (Mills et al., 2000). Polarized macrophages perform different roles in immunoregulation, inflammation, tissue remodeling, proliferation, and metabolism. Among them, M1 macrophages are key effector cells against intracellular pathogens (Hill et al., 2014; Zanganeh et al., 2016), while M2 macrophages can promote tissue remodeling (Biswas et al., 2012).

Nanomaterials, as a stimulant, can promote the initiation of macrophages to different polarization states in the microenvironment (Lucarelli et al., 2004; Bartneck et al., 2012; Laskar et al., 2013; Fuchs et al., 2016). Available data suggest that the ability of nanomaterials to regulate M1 polarization is influenced by their physicochemical properties, such as chemical composition, size, and surface coatings (Lucarelli et al., 2004; Yen et al., 2010; Tran et al., 2015).

For instance, metal NPs such as Au NPs and Ag NPs can directly induce M1 polarization, but Au NPs have a greater effect than Ag NPs in inducing M1 polarization (Yen et al., 2010). Other studies have shown that the pro-inflammatory effects of Ag, TiO_2_, and ZnO NPS in RAW264.7 macrophages are related to their doses in ultralow concentration (Miao et al., 2017). Besides, compared with the inert ceramic NPs (such as TiO_2_ NPs and ZrO_2_ NPs), SiO_2_ NPs are more likely to stimulate primary macrophages toward a pro-inflammatory M1 subtype (Lucarelli et al., 2004).

Graphene oxide (GO) NPs, in a size-dependent manner, induced macrophages to polarize the M1 phenotype (Ma et al., 2015), with large GO nanosheets inducing a higher production of inflammatory cytokines than smaller ones. However, it was reported that for most metallic NPs (Ag, Al, and Au NP), smaller size NPs have greater effects in inducing M1 macrophage polarization than their larger counterparts (Yen et al., 2010; Nishanth et al., 2011).

Surface modifications of bioactive peptides regulate macrophages toward contrasting polarization states. Au nanorod–modified glycine–leucine–phenylalanine (GLF) is more pro-inflammatory than arginine–glycine–aspartic acid (RGD) Au nanorods, directing isolated hepatic macrophages to the M1 subtype (Bartneck et al., 2012).

In addition, when stimuli change, M1 macrophages can be re-educated into the M2 phenotype, and vice versa. This process is referred to as macrophage reprogramming or repolarization (Jain et al., 2015; Lee et al., 2016). Superparamagnetic iron-oxide nanoparticles (SPIONs) could shift macrophages from the M2 to M1, by changing the cellular iron concentration. Glycocalyx-mimicking NPs (glycol-NPs) were observed to reverse the M2 phenotype, skewing the mouse peritoneal macrophage–derived M2 phenotype into an M1 phenotype (Su et al., 2015). Polystyrene NPs with surface carboxyl and amino groups could strongly skew the M2 macrophage polarization without affecting M1 markers (Fuchs et al., 2016).

## Challenges and Prospects

Nanomaterials have numerous advantageous features in the biomedical field that promise in addressing the key hurdles in treating biofilm infection . Regrettably, there are still challenges that need to be resolved urgently to be translated to clinical application.

Chemists have probably created a number of nanotechnology-based antimicrobials that promise to face the tricky infectious biofilms. However, partly driven by the motivation of researchers to write high-impact articles, few of them are translated to the bedside for the benefit of patients (Liu et al., 2019). As a consequence, how to achieve commercialization and clinical application with low cost and high efficiency will be the primary problem faced by the antibacterial nanomaterials.

In addition, considering *in vivo* conditions, such as blood and host immune components, tissue cells, and interactions between bacteria and antibiotics, the results obtained from *in vitro* experiments are difficult to be applied directly *in vivo* (Mahmoudi et al., 2009; Mahmoudi et al., 2010). Even if some experiments have been carried out related to animal research and achieved relatively satisfactory results, whether the results of animal experiments can be replicated in humans has to be identified, due to the physiological differences between human and animals. And in clinical microbiology, statistically significant differences of 2–10 or even 100 times are meaningless (Liu et al., 2019). The clinical significance begins with a reduction of at least 3 to 4 logarithms of the viable count (representing a 99.9 and 99.99% decrease, respectively), while in the field of the development of new antimicrobial strategies based on nanotechnology, many articles report statistically significant reductions of less than 1 logarithmic unit, or only 90% (Nguyen et al., 2016), which is microbiologically insignificant. It cannot be used as a primary parameter to represent clinical benefits, which are defined by the proportion of patients cured by a new drug compared with existing treatment and costs.

Intravenous nanomaterials have been shown to accumulate in the colon, lung, bone marrow, liver, spleen, and lymph nodes (Hagens et al., 2007), while inhaled nanomaterials can be efficiently absorbed by epithelial and endothelial cells entering the blood and lymphatic circulation to reach the lungs, liver, heart, spleen, and other organs (Rabea et al., 2003; Poma and Di Giorgio, 2008). Currently, the potential toxicity of antibiotic nanoparticles to human health is poorly understood, although many studies have suggested that therapeutic nanoparticles may produce multiple organ toxicity (Poma and Di Giorgio, 2008). For example, free radical–mediated oxidative stress resulting from the interaction of antimicrobial nanoparticles with cells may lead to hepatotoxicity and pulmonary toxicity (De Jong and Borm, 2008; Lei et al., 2008). Various metabolic changes indicate mitochondrial failure, enhanced ketone production, fatty acid *β*-oxidation, and glycolysis, leading to hepatotoxicity and nephrotoxicity (Lei et al., 2008). Antibacterial nanoparticles also have interaction with the central nervous system, but their toxicity is unknown (Hu and Gao, 2010). In addition, certain classes of NP can affect the reproductive system by increasing sperm epithelial separation and possible sperm toxicity (Komatsu et al., 2008; Yoshida et al., 2010). Nanoparticles may degrade metabolism and excrete through renal/fecal matter (Corot et al., 2006), for example, iron oxide nanoparticles, whereas the body does not possess mechanisms to process heavier elements and some nanoparticles (Naha et al., 2016), such as gold. A profound knowledge of nanomaterials’ potential toxicity is needed, including a comprehensive assessment of the interactions with cells, tissues, and organs in order to recalibrate doses, determine appropriate routes of administration, and develop relevant test criteria to achieve the desired clinical translation (Suri et al., 2007; Sandhiya et al., 2009).

## Conclusion

In short, nanomaterials are promising delivery vehicles and can themselves act as antimicrobial agents, whose ability to penetrate biofilms also holds promise as a treatment for particularly hard-to-treat infections. However, there is still a long way to go before successful clinical translation, which requires the joint efforts of multiple disciplines.

## References

[B1] Aflori M. (2021). Smart Nanomaterials for Biomedical Applications-A Review. Nanomaterials 11 (2), 396. 10.3390/nano11020396 33557177 PMC7913901

[B2] Albayaty Y. N. Thomas N. Jambhrunkar M. Al-Hawwas M. Kral A. Thorn C. R. (2019). Enzyme Responsive Copolymer Micelles Enhance the Anti-biofilm Efficacy of the Antiseptic Chlorhexidine. Int. J. Pharmaceutics 566, 329–341. 10.1016/j.ijpharm.2019.05.069 31152793

[B3] Anderson J. M. (2016). Future Challenges in Thein Vitroandin Vivoevaluation of Biomaterial Biocompatibility. Regen. Biomater. 3 (2), 73–77. 10.1093/rb/rbw001 27047672 PMC4817327

[B4] Arciola C. R. Campoccia D. Montanaro L. (2018). Implant Infections: Adhesion, Biofilm Formation and Immune Evasion. Nat. Rev. Microbiol. 16 (7), 397–409. 10.1038/s41579-018-0019-y 29720707

[B5] Baptista P. V. McCusker M. P. Carvalho A. Ferreira D. A. Mohan N. M. Martins M. (2018). Nano-Strategies to Fight Multidrug Resistant Bacteria-"A Battle of the Titans". Front. Microbiol. 9, 1441. 10.3389/fmicb.2018.01441 30013539 PMC6036605

[B6] Bartneck M. Ritz T. Keul H. A. Wambach M. Bornemann J. Gbureck U. (2012). Peptide-functionalized Gold Nanorods Increase Liver Injury in Hepatitis. Acs Nano 6 (10), 8767–8777. 10.1021/nn302502u 22994679

[B7] Benoit D. S. W. Sims K. R. Jr. Fraser D. (2019). Nanoparticles for Oral Biofilm Treatments. ACS Nano 13 (5), 4869–4875. 10.1021/acsnano.9b02816 31033283 PMC6707515

[B8] Biswas S. K. Chittezhath M. Shalova I. N. Lim J.-Y. (2012). Macrophage Polarization and Plasticity in Health and Disease. Immunol. Res. 53 (1-3), 11–24. 10.1007/s12026-012-8291-9 22418728

[B9] Brown A. A. Azzaroni O. Huck W. T. S. (2009). Photoresponsive Polymer Brushes for Hydrophilic Patterning. Langmuir 25 (3), 1744–1749. 10.1021/la8032308 19132832

[B10] Chen M. Wei J. Xie S. Tao X. Zhang Z. Ran P. (2019). Bacterial Biofilm Destruction by Size/surface Charge-Adaptive Micelles. Nanoscale 11 (3), 1410–1422. 10.1039/c8nr05575k 30608101

[B11] Childs S. G. (2008). Biofilm. Orthop. Nurs. 27 (6), 361–369. quiz 370-361. 10.1097/01.NOR.0000342424.51960.ce 19057364

[B12] Ciofu O. (2003). *Pseudomonas aeruginosa* Chromosomal Beta-Lactamase in Patients with Cystic Fibrosis and Chronic Lung Infection. Mechanism of Antibiotic Resistance and Target of the Humoral Immune Response. APMIS Suppl. Suppl (116), 1–47. 14692154

[B13] Corot C. Robert P. Idée J. Port M. (2006). Recent Advances in Iron Oxide Nanocrystal Technology for Medical Imaging☆. Adv. Drug Deliv. Rev. 58 (14), 1471–1504. 10.1016/j.addr.2006.09.013 17116343

[B14] Costerton J. W. (2005). Biofilm Theory Can Guide the Treatment of Device-Related Orthopaedic Infections. Clin. Orthopaedics Relat. Res. & NA, 7–11. 10.1097/00003086-200508000-00003 16056019

[B15] Costerton J. W. Geesey G. G. Cheng K.-J. (1978). How Bacteria Stick. Sci. Am. 238 (1), 86–95. 10.1038/scientificamerican0178-86 635520

[B16] Date A. A. Hanes J. Ensign L. M. (2016). Nanoparticles for Oral Delivery: Design, Evaluation and State-Of-The-Art. J. Controlled Release 240, 504–526. 10.1016/j.jconrel.2016.06.016 PMC5064878 27292178

[B17] De Jong W. H. Borm P. J. (2008). Drug Delivery and Nanoparticles: Applications and Hazards. Ijn 3 (2), 133–149. 10.2147/ijn.s596 18686775 PMC2527668

[B18] Ding Y. Hao Y. Yuan Z. Tao B. Chen M. Lin C. (2020). A Dual-Functional Implant with an Enzyme-Responsive Effect for Bacterial Infection Therapy and Tissue Regeneration. Biomater. Sci. 8 (7), 1840–1854. 10.1039/c9bm01924c 31967110

[B19] Drancourt M. Stein A. Argenson J. N. Zannier A. Curvale G. Raoult D. (1993). Oral Rifampin Plus Ofloxacin for Treatment of Staphylococcus-Infected Orthopedic Implants. Antimicrob. Agents Chemother. 37 (6), 1214–1218. 10.1128/AAC.37.6.1214 8328772 PMC187942

[B20] Driffield K. Miller K. Bostock J. M. O'Neill A. J. Chopra I. (2008). Increased Mutability of *Pseudomonas aeruginosa* in Biofilms. J. Antimicrob. Chemother. 61 (5), 1053–1056. 10.1093/jac/dkn044 18256114

[B21] Ehrlich G. D. Stoodley P. Kathju S. Zhao Y. McLeod B. R. Balaban N. (2005). Engineering Approaches for the Detection and Control of Orthopaedic Biofilm Infections. Clin. Orthopaedics Relat. Res. & NA, 59–66. 10.1097/00003086-200508000-00011 PMC1351327 16056027

[B22] Elbourne A. Cheeseman S. Atkin P. Truong N. P. Syed N. Zavabeti A. (2020). Antibacterial Liquid Metals: Biofilm Treatment via Magnetic Activation. ACS Nano 14 (1), 802–817. 10.1021/acsnano.9b07861 31922722

[B23] Eleraky N. E. Allam A. Hassan S. B. Omar M. M. (2020). Nanomedicine Fight against Antibacterial Resistance: An Overview of the Recent Pharmaceutical Innovations. Pharmaceutics 12 (2), 142. 10.3390/pharmaceutics12020142 PMC7076477 32046289

[B24] Espeland E. M. Wetzel R. G. (2001). Complexation, Stabilization, and UV Photolysis of Extracellular and Surface-Bound Glucosidase and Alkaline Phosphatase: Implications for Biofilm Microbiota. Microb. Ecol. 42 (4), 572–585. 10.1007/s00248-001-1023-7 12024240

[B25] Flemming H.-C. Neu T. R. Wozniak D. J. (2007). The EPS Matrix: the "house of Biofilm Cells". J. Bacteriol. 189 (22), 7945–7947. 10.1128/jb.00858-07 17675377 PMC2168682

[B26] Flemming H.-C. Wingender J. (2010). The Biofilm Matrix. Nat. Rev. Microbiol. 8 (9), 623–633. 10.1038/nrmicro2415 20676145

[B27] Flores-Mireles A. L. Walker J. N. Caparon M. Hultgren S. J. (2015). Urinary Tract Infections: Epidemiology, Mechanisms of Infection and Treatment Options. Nat. Rev. Microbiol. 13 (5), 269–284. 10.1038/nrmicro3432 25853778 PMC4457377

[B28] Fuchs A.-K. Syrovets T. Haas K. A. Loos C. Musyanovych A. Mailänder V. (2016). Carboxyl- and Amino-Functionalized Polystyrene Nanoparticles Differentially Affect the Polarization Profile of M1 and M2 Macrophage Subsets. Biomaterials 85, 78–87. 10.1016/j.biomaterials.2016.01.064 26854393

[B29] Gbejuade H. O. Lovering A. M. Webb J. C. (2015). The Role of Microbial Biofilms in Prosthetic Joint Infections. Acta Orthopaedica 86 (2), 147–158. 10.3109/17453674.2014.966290 25238433 PMC4404764

[B30] Genevaux P. Muller S. Bauda P. (1996). A Rapid Screening Procedure to Identify Mini-Tn10 Insertion Mutants ofEscherichia coliK-12 with Altered Adhesion Properties. FEMS Microbiol. Lett. 142 (1), 27–30. 10.1111/j.1574-6968.1996.tb08402.x 8759786

[B31] Hagens W. I. Oomen A. G. de Jong W. H. Cassee F. R. Sips A. J. A. M. (2007). What Do We (Need to) Know about the Kinetic Properties of Nanoparticles in the Body? Regul. Toxicol. Pharmacol. 49 (3), 217–229. 10.1016/j.yrtph.2007.07.006 17868963

[B32] Hall-Stoodley L. Costerton J. W. Stoodley P. (2004). Bacterial Biofilms: from the Natural Environment to Infectious Diseases. Nat. Rev. Microbiol. 2 (2), 95–108. 10.1038/nrmicro821 15040259

[B33] Halwani M. Mugabe C. Azghani A. O. Lafrenie R. M. Kumar A. Omri A. (2007). Bactericidal Efficacy of Liposomal Aminoglycosides against Burkholderia Cenocepacia. J. Antimicrob. Chemother. 60 (4), 760–769. 10.1093/jac/dkm289 17673475

[B34] Hill A. A. Reid Bolus W. Hasty A. H. (2014). A Decade of Progress in Adipose Tissue Macrophage Biology. Immunol. Rev. 262 (1), 134–152. 10.1111/imr.12216 25319332 PMC4203421

[B35] Høiby N. Bjarnsholt T. Givskov M. Molin S. Ciofu O. (2010). Antibiotic Resistance of Bacterial Biofilms. Int. J. Antimicrob. Agents 35 (4), 322–332. 10.1016/j.ijantimicag.2009.12.011 20149602

[B36] Høiby N. Bjarnsholt T. Moser C. Bassi G. L. Coenye T. Donelli G. (2015). ESCMID∗ Guideline for the Diagnosis and Treatment of Biofilm Infections 2014. Clin. Microbiol. Infect. 21 (Suppl. 1), S1–S25. 10.1016/j.cmi.2014.10.024 25596784

[B37] Horev B. Klein M. I. Hwang G. Li Y. Kim D. Koo H. (2015). pH-activated Nanoparticles for Controlled Topical Delivery of Farnesol to Disrupt Oral Biofilm Virulence. Acs Nano 9 (3), 2390–2404. 10.1021/nn507170s 25661192 PMC4395463

[B38] Hossion A. M. L. Bio M. Nkepang G. Awuah S. G. You Y. (2013). Visible Light Controlled Release of Anticancer Drug through Double Activation of Prodrug. ACS Med. Chem. Lett. 4 (1), 124–127. 10.1021/ml3003617 24900573 PMC4027153

[B39] Hu D. Deng Y. Jia F. Jin Q. Ji J. (2020). Surface Charge Switchable Supramolecular Nanocarriers for Nitric Oxide Synergistic Photodynamic Eradication of Biofilms. ACS Nano 14 (1), 347–359. 10.1021/acsnano.9b05493 31887012

[B40] Hu D. Li H. Wang B. Ye Z. Lei W. Jia F. (2017). Surface-Adaptive Gold Nanoparticles with Effective Adherence and Enhanced Photothermal Ablation of Methicillin-ResistantStaphylococcus aureusBiofilm. ACS Nano 11 (9), 9330–9339. 10.1021/acsnano.7b04731 28806528

[B41] Hu Y.-L. Gao J.-Q. (2010). Potential Neurotoxicity of Nanoparticles. Int. J. Pharmaceutics 394 (1-2), 115–121. 10.1016/j.ijpharm.2010.04.026 20433914

[B42] Jain S. Tran T.-H. Amiji M. (2015). Macrophage Repolarization with Targeted Alginate Nanoparticles Containing IL-10 Plasmid DNA for the Treatment of Experimental Arthritis. Biomaterials 61, 162–177. 10.1016/j.biomaterials.2015.05.028 26004232 PMC4464978

[B43] Joshi A. S. Singh P. Mijakovic I. (2020). Interactions of Gold and Silver Nanoparticles with Bacterial Biofilms: Molecular Interactions behind Inhibition and Resistance. Ijms 21 (20), 7658. 10.3390/ijms21207658 PMC7589962 33081366

[B44] Karimi M. Ghasemi A. Sahandi Zangabad P. Rahighi R. Moosavi Basri S. M. Mirshekari H. (2016). Smart Micro/nanoparticles in Stimulus-Responsive Drug/gene Delivery Systems. Chem. Soc. Rev. 45 (5), 1457–1501. 10.1039/c5cs00798d 26776487 PMC4775468

[B45] Karol M. N. Hamilton I. R. (2010). Acid Tolerance Response of Biofilm Cells of Streptococcus Mutans. Fems Microbiol. Lett. (1), 25–30. 10.1016/S0378-1097(03)00164-2 12694906

[B46] Karthikeyan T. G. Kondareddy C. Hwangjae L. Yeon J. Y. In-Kyu P. Young L. J. (2018). Magnetic Field-Inducible Drug-Eluting Nanoparticles for Image-Guided Thermo-Chemotherapy. Biomaterials 180, 240–252. 10.1016/j.biomaterials.2018.07.028 30055399

[B47] Khan F. Lee J.-W. Pham D. N. T. Khan M. M. Park S.-K. Shin I.-S. (2020a). Antibiofilm Action of ZnO, SnO2 and CeO2 Nanoparticles towards Grampositive Biofilm Forming Pathogenic Bacteria. Nanotec 14 (3), 239–249. 10.2174/1872210514666200313121953 32167434

[B48] Khan F. Park S.-K. Bamunuarachchi N. I. Oh D. Kim Y.-M. (2021). Caffeine-loaded Gold Nanoparticles: Antibiofilm and Anti-persister Activities against Pathogenic Bacteria. Appl. Microbiol. Biotechnol. 105 (9), 3717–3731. 10.1007/s00253-021-11300-3 33900427

[B49] Khan F. Tabassum N. Kim Y.-M. (2020b). A Strategy to Control Colonization of Pathogens: Embedding of Lactic Acid Bacteria on the Surface of Urinary Catheter. Appl. Microbiol. Biotechnol. 104 (21), 9053–9066. 10.1007/s00253-020-10903-6 32949279

[B50] Khan F. Yu H. Kim Y.-M. (2020c). Bactericidal Activity of Usnic Acid-Chitosan Nanoparticles against Persister Cells of Biofilm-Forming Pathogenic Bacteria. Mar. Drugs 18 (5), 270. 10.3390/md18050270 PMC7281555 32443816

[B51] Kim M.-H. Yamayoshi I. Mathew S. Lin H. Nayfach J. Simon S. I. (2013). Magnetic Nanoparticle Targeted Hyperthermia of Cutaneous *Staphylococcus aureus* Infection. Ann. Biomed. Eng. 41 (3), 598–609. 10.1007/s10439-012-0698-x 23149904 PMC3740557

[B52] Komatsu T. Tabata M. Kubo-Irie M. Shimizu T. Suzuki K.-i. Nihei Y. (2008). The Effects of Nanoparticles on Mouse Testis Leydig Cells *In Vitro* . Toxicol. Vitro 22 (8), 1825–1831. 10.1016/j.tiv.2008.08.009 18805477

[B53] Koo H. Allan R. N. Howlin R. P. Stoodley P. Hall-Stoodley L. (2017). Targeting Microbial Biofilms: Current and Prospective Therapeutic Strategies. Nat. Rev. Microbiol. 15 (12), 740–755. 10.1038/nrmicro.2017.99 28944770 PMC5685531

[B54] Kumar A. Alam A. Grover S. Pandey S. Tripathi D. Kumari M. (2019). Peptidyl-prolyl Isomerase-B Is Involved in *Mycobacterium tuberculosis* Biofilm Formation and a Generic Target for Drug Repurposing-Based Intervention. NPJ Biofilms Microbiomes 5 (1), 3. 10.1038/s41522-018-0075-0 30675370 PMC6333787

[B55] Kumar M. Curtis A. Hoskins C. (2018). Application of Nanoparticle Technologies in the Combat against Anti-microbial Resistance. Pharmaceutics 10 (1), 11. 10.3390/pharmaceutics10010011 PMC5874824 29342903

[B56] Laskar A. Eilertsen J. Li W. Yuan X.-M. (2013). SPION Primes THP1 Derived M2 Macrophages towards M1-like Macrophages. Biochem. Biophysical Res. Commun. 441 (4), 737–742. 10.1016/j.bbrc.2013.10.115 24184477

[B57] Le Magrex-Debar E. Lemoine J. Gellé M. P. Jacquelin L. F. Choisy C. (2000). Evaluation of Biohazards in Dehydrated Biofilms on Foodstuff Packaging. Int. J. Food Microbiol. 55 (1-3), 239–243. 10.1016/s0168-1605(00)00177-x 10791750

[B58] Lebeaux D. Ghigo J.-M. Beloin C. (2014). Biofilm-related Infections: Bridging the gap between Clinical Management and Fundamental Aspects of Recalcitrance toward Antibiotics. Microbiol. Mol. Biol. Rev. 78 (3), 510–543. 10.1128/mmbr.00013-14 25184564 PMC4187679

[B59] Lee C.-H. Kim Y.-J. Jang J.-H. Park J.-W. (2016). Modulating Macrophage Polarization with Divalent Cations in Nanostructured Titanium Implant Surfaces. Nanotechnology 27 (8), 085101. 10.1088/0957-4484/27/8/085101 26807875

[B60] Lei R. Wu C. Yang B. Ma H. Shi C. Wang Q. (2008). Integrated Metabolomic Analysis of the Nano-Sized Copper Particle-Induced Hepatotoxicity and Nephrotoxicity in Rats: a Rapid *In Vivo* Screening Method for Nanotoxicity. Toxicol. Appl. Pharmacol. 232 (2), 292–301. 10.1016/j.taap.2008.06.026 18706438

[B61] Leid J. G. Shirtliff M. E. Costerton J. W. Stoodley a. P. (2002). Human Leukocytes Adhere to, Penetrate, and Respond to *Staphylococcus aureus* Biofilms. Infect. Immun. 70 (11), 6339–6345. 10.1128/iai.70.11.6339-6345.2002 12379713 PMC130380

[B62] Lewis K. (2008). Multidrug Tolerance of Biofilms and Persister Cells. Curr. Top. Microbiol. Immunol. 322, 107–131. 10.1007/978-3-540-75418-3_6 18453274

[B63] Lewis K. (2001). Riddle of Biofilm Resistance. Antimicrob. Agents Chemother. 45 (4), 999–1007. 10.1128/aac.45.4.999-1007.2001 11257008 PMC90417

[B64] Li C. Zhang X. Xinliang X. Xiaoying X. Guojian G. Chen Z. (2013). Preparation and Characterization of Flexible Nanoliposomes Loaded with Daptomycin, a Novel Antibiotic, for Topical Skin Therapy. Ijn 8, 1285–1292. 10.2147/ijn.S41695 23569376 PMC3615926

[B65] Li J. Nickel R. Wu J. Lin F. van Lierop J. Liu S. (2019a). A New Tool to Attack Biofilms: Driving Magnetic Iron-Oxide Nanoparticles to Disrupt the Matrix. Nanoscale 11 (14), 6905–6915. 10.1039/c8nr09802f 30912773

[B66] Li L.-L. Yu P. Wang X. Yu S.-S. Mathieu J. Yu H.-Q. (2017). Enhanced Biofilm Penetration for Microbial Control by Polyvalent Phages Conjugated with Magnetic Colloidal Nanoparticle Clusters (CNCs). Environ. Sci. Nano 4 (9), 1817–1826. 10.1039/C7EN00414A

[B67] Li P. Liu S. Zhang G. Yang X. Cao W. Gong X. (2020a). Design of pH-Responsive Dissociable Nanosystem Based on Carbon Dots with Enhanced Anti-biofilm Property and Excellent Biocompatibility. ACS Appl. Bio Mater. 3 (2), 1105–1115. 10.1021/acsabm.9b01053 35019312

[B68] Li Q. Li W. Di H. Luo L. Zhu C. Yang J. (2018). A Photosensitive Liposome with NIR Light Triggered Doxorubicin Release as a Combined Photodynamic-Chemo Therapy System. J. Controlled Release 277, 114–125. 10.1016/j.jconrel.2018.02.001 29408424

[B69] Li S. Chang R. Chen J. Mi G. Xie Z. Webster T. J. (2020b). Novel Magnetic Nanocomposites Combining Selenium and Iron Oxide with Excellent Anti-biofilm Properties. J. Mater. Sci. 55 (3), 1012–1022. 10.1007/s10853-019-04019-0

[B70] Li W. Geng X. Liu D. Li Z. (2019b). Near-Infrared Light-Enhanced Protease-Conjugated Gold Nanorods as A Photothermal Antimicrobial Agent for Elimination of Exotoxin and Biofilms. Ijn Vol. 14, 8047–8058. 10.2147/ijn.S212750 PMC6781946 31632017

[B71] Lin J. Hu J. Wang W. Liu K. Zhou C. Liu Z. (2021). Thermo and Light-Responsive Strategies of Smart Titanium-Containing Composite Material Surface for Enhancing Bacterially Anti-adhesive Property. Chem. Eng. J. 407, 125783. 10.1016/j.cej.2020.125783

[B72] Liu C. Zhang Y. Liu M. Chen Z. Lin Y. Li W. (2017). A NIR-Controlled Cage Mimicking System for Hydrophobic Drug Mediated Cancer Therapy. Biomaterials 139, 151–162. 10.1016/j.biomaterials.2017.06.008 28618345

[B73] Liu Y. Busscher H. J. Zhao B. Li Y. Zhang Z. van der Mei H. C. (2016). Surface-Adaptive, Antimicrobially Loaded, Micellar Nanocarriers with Enhanced Penetration and Killing Efficiency in Staphylococcal Biofilms. ACS Nano 10 (4), 4779–4789. 10.1021/acsnano.6b01370 26998731

[B74] Liu Y. Shi L. Su L. van der Mei H. C. Jutte P. C. Ren Y. (2019). Nanotechnology-based Antimicrobials and Delivery Systems for Biofilm-Infection Control. Chem. Soc. Rev. 48 (2), 428–446. 10.1039/c7cs00807d 30601473

[B75] Lucarelli M. Gatti A. M. Savarino G. Quattroni P. Martinelli L. Monari E. (2004). Innate Defence Functions of Macrophages Can Be Biased by Nano-Sized Ceramic and Metallic Particles. Eur. Cytokine Netw. 15 (4), 339–346. 15627643

[B76] Ma J. Liu R. Wang X. Liu Q. Chen Y. Valle R. P. (2015). Crucial Role of Lateral Size for Graphene Oxide in Activating Macrophages and Stimulating Pro-inflammatory Responses in Cells and Animals. Acs Nano 9 (10), 10498–10515. 10.1021/acsnano.5b04751 26389709 PMC5522963

[B77] Mahmoudi M. Serpooshan V. (2012). Silver-Coated Engineered Magnetic Nanoparticles Are Promising for the Success in the Fight against Antibacterial Resistance Threat. Acs Nano 6 (3), 2656–2664. 10.1021/nn300042m 22397679

[B78] Mahmoudi M. Simchi A. Imani M. Milani A. S. Stroeve P. (2009). Anin Vitrostudy of Bare and Poly(ethylene Glycol)-Co-Fumarate-Coated Superparamagnetic Iron Oxide Nanoparticles: a New Toxicity Identification Procedure. Nanotechnology 20 (22), 225104. 10.1088/0957-4484/20/22/225104 19433870

[B79] Mahmoudi M. Simchi A. Imani M. Shokrgozar M. A. Milani A. S. Häfeli U. O. (2010). A New Approach for the *In Vitro* Identification of the Cytotoxicity of Superparamagnetic Iron Oxide Nanoparticles. Colloids Surf. B: Biointerfaces 75 (1), 300–309. 10.1016/j.colsurfb.2009.08.044 19781921

[B80] Makabenta J. M. V. Nabawy A. Li C.-H. Schmidt-Malan S. Patel R. Rotello V. M. (2021). Nanomaterial-based Therapeutics for Antibiotic-Resistant Bacterial Infections. Nat. Rev. Microbiol. 19 (1), 23–36. 10.1038/s41579-020-0420-1 32814862 PMC8559572

[B81] Matthew S. Ajay P. Sheth N. P. (2018). Projected Volume of Primary Total Joint Arthroplasty in the U.S., 2014 to 2030. JBJS 100 (17), 1455–1460. 10.2106/JBJS.17.01617 30180053

[B82] Menkin V. (1931). Studies on Inflammation. J. Exp. Med. 53 (5), 647–660. 10.1084/jem.53.5.647 19869871 PMC2131987

[B83] Miao X. Leng X. Zhang Q. (2017). The Current State of Nanoparticle-Induced Macrophage Polarization and Reprogramming Research. Ijms 18 (2), 336. 10.3390/ijms18020336 PMC5343871 28178185

[B84] Mills C. D. Kincaid K. Alt J. M. Heilman M. J. Hill A. M. (2000). M-1/M-2 Macrophages and the Th1/Th2 Paradigm. J. Immunol. 164 (12), 6166–6173. 10.4049/jimmunol.164.12.6166 10843666

[B85] Mugabe C. Halwani M. Azghani A. O. Lafrenie R. M. Omri A. (2006). Mechanism of Enhanced Activity of Liposome-Entrapped Aminoglycosides against Resistant Strains of *Pseudomonas aeruginosa* . Antimicrob. Agents Chemother. 50 (6), 2016–2022. 10.1128/aac.01547-05 16723560 PMC1479138

[B86] Naha P. C. Lau K. C. Hsu J. C. Hajfathalian M. Mian S. Chhour P. (2016). Gold Silver alloy Nanoparticles (GSAN): an Imaging Probe for Breast Cancer Screening with Dual-Energy Mammography or Computed Tomography. Nanoscale 8 (28), 13740–13754. 10.1039/c6nr02618d 27412458 PMC4955565

[B87] Nguyen T.-K. Selvanayagam R. Ho K. K. K. Chen R. Kutty S. K. Rice S. A. (2016). Co-delivery of Nitric Oxide and Antibiotic Using Polymeric Nanoparticles. Chem. Sci. 7 (2), 1016–1027. 10.1039/c5sc02769a 28808526 PMC5531038

[B88] Nickel J. C. Ruseska I. Wright J. B. Costerton J. W. (1985). Tobramycin Resistance of *Pseudomonas aeruginosa* Cells Growing as a Biofilm on Urinary Catheter Material. Antimicrob. Agents Chemother. 27 (4), 619–624. 10.1128/aac.27.4.619 3923925 PMC180108

[B89] Nishanth R. P. Jyotsna R. G. Schlager J. J. Hussain S. M. Reddanna P. (2011). Inflammatory Responses of RAW 264.7 Macrophages upon Exposure to Nanoparticles: Role of ROS-Nfκb Signaling Pathway. Nanotoxicology 5 (4), 502–516. 10.3109/17435390.2010.541604 21417802

[B90] Nistico L. Hall-Stoodley L. Stoodley P. (2014). Imaging Bacteria and Biofilms on Hardware and Periprosthetic Tissue in Orthopedic Infections. Methods Mol. Biol. 1147 (1147), 105–126. 10.1007/978-1-4939-0467-9_8 24664829

[B91] O'Toole G. A. Kolter R. (1998). Flagellar and Twitching Motility Are Necessary forPseudomonas Aeruginosabiofilm Development. Mol. Microbiol. 30 (2), 295–304. 10.1046/j.1365-2958.1998.01062.x 9791175

[B92] Pamp S. J. Gjermansen M. Johansen H. K. Tolker-Nielsen T. (2008). Tolerance to the Antimicrobial Peptide Colistin in *Pseudomonas aeruginosa* Biofilms Is Linked to Metabolically Active Cells, and Depends on the Pmr and mexAB-oprM Genes. Mol. Microbiol. 68 (1), 223–240. 10.1111/j.1365-2958.2008.06152.x 18312276

[B93] Park H. Park H.-J. Kim J. A. Lee S. H. Kim J. H. Yoon J. (2011). Inactivation of *Pseudomonas aeruginosa* PA01 Biofilms by Hyperthermia Using Superparamagnetic Nanoparticles. J. Microbiol. Methods 84 (1), 41–45. 10.1016/j.mimet.2010.10.010 20971135

[B94] Pham S. H. Choi Y. Choi J. (2020). Stimuli-Responsive Nanomaterials for Application in Antitumor Therapy and Drug Delivery. Pharmaceutics 12 (7), 630. 10.3390/pharmaceutics12070630 PMC7408499 32635539

[B95] Poma A. Di Giorgio M. (2008). Toxicogenomics to Improve Comprehension of the Mechanisms Underlying Responses of *In Vitro* and *In Vivo* Systems to Nanomaterials: a Review. Cg 9 (8), 571–585. 10.2174/138920208786847962 PMC2694561 19516964

[B96] Pornpattananangkul D. Zhang L. Olson S. Aryal S. Obonyo M. Vecchio K. (2011). Bacterial Toxin-Triggered Drug Release from Gold Nanoparticle-Stabilized Liposomes for the Treatment of Bacterial Infection. J. Am. Chem. Soc. 133 (11), 4132–4139. 10.1021/ja111110e 21344925 PMC3062754

[B97] Pratt L. A. Kolter R. (1998). Genetic Analysis ofEscherichia Colibiofilm Formation: Roles of Flagella, Motility, Chemotaxis and Type I Pili. Mol. Microbiol. 30 (2), 285–293. 10.1046/j.1365-2958.1998.01061.x 9791174

[B98] Qayyum S. Khan A. U. (2016). Nanoparticles vs. Biofilms: a Battle against Another Paradigm of Antibiotic Resistance. Med. Chem. Commun. 7 (8), 1479–1498. 10.1039/C6MD00124F

[B99] Rabea E. I. Badawy M. E.-T. Stevens C. V. Smagghe G. Steurbaut W. (2003). Chitosan as Antimicrobial Agent: Applications and Mode of Action. Biomacromolecules 4 (6), 1457–1465. 10.1021/bm034130m 14606868

[B100] Raju P. Arivalagan P. Natarajan S. (2020). One-pot Fabrication of Multifunctional Catechin@ZIF-L Nanocomposite: Assessment of Antibiofilm, Larvicidal and Photocatalytic Activities. J. Photochem. Photobiol. B: Biol. 203, 111774. 10.1016/j.jphotobiol.2019.111774 31931386

[B101] Raza A. Rasheed T. Nabeel F. Hayat U. Bilal M. Iqbal H. (2019). Endogenous and Exogenous Stimuli-Responsive Drug Delivery Systems for Programmed Site-specific Release. Molecules 24 (6), 1117. 10.3390/molecules24061117 PMC6470858 30901827

[B102] Rodríguez R. Zamora J. M. Salinasrodríguez E. Izquierdo E. (2011). Stochastic Modeling of Some Aspects of Biofilm Behavior. Revista Mexicana De Fisica 49 (2), 132–143.

[B103] Sandhiya S. Dkhar S. A. Surendiran A. (2009). Emerging Trends of Nanomedicine - an Overview. Fundam. Clin. Pharmacol. 23 (3), 263–269. 10.1111/j.1472-8206.2009.00692.x 19527298

[B104] Schinsky M. F. Della Valle C. J. Sporer S. M. Paprosky W. G. (2008). Perioperative Testing for Joint Infection in Patients Undergoing Revision Total Hip Arthroplasty. The J. Bone Jt. Surgery-American Volume 90 (9), 1869–1875. 10.2106/jbjs.G.01255 18762646

[B105] Schleich N. Danhier F. Préat V. (2015). Iron Oxide-Loaded Nanotheranostics: Major Obstacles to *In Vivo* Studies and Clinical Translation. J. Controlled Release 198, 35–54. 10.1016/j.jconrel.2014.11.024 25481448

[B106] Schmitt J. Flemming H.-C. (1999). Water Binding in Biofilms. Water Sci. Technology 39 (7), 77–82. 10.2166/wst.1999.0333

[B107] Shoji M. M. Chen A. F. (2020). Biofilms in Periprosthetic Joint Infections: A Review of Diagnostic Modalities, Current Treatments, and Future Directions. J. Knee Surg. 33 (2), 119–131. 10.1055/s-0040-1701214 31935760

[B108] Shuman E. K. Chenoweth C. E. (2018). Urinary Catheter-Associated Infections. Infect. Dis. Clin. North America 32 (4), 885–897. 10.1016/j.idc.2018.07.002 30241712

[B109] Stewart P. S. William Costerton J. (2001). Antibiotic Resistance of Bacteria in Biofilms. The Lancet 358 (9276), 135–138. 10.1016/s0140-6736(01)05321-1 11463434

[B110] Su L. Zhang W. Wu X. Zhang Y. Chen X. Liu G. (2015). Glycocalyx-Mimicking Nanoparticles for Stimulation and Polarization of Macrophages via Specific Interactions. Small 11 (33), 4191–4200. 10.1002/smll.201403838 25994111

[B111] Suri S. Fenniri H. Singh B. (2007). Nanotechnology-based Drug Delivery Systems. J. Occup. Med. Toxicol. 2, 16. 10.1186/1745-6673-2-16 18053152 PMC2222591

[B112] Sutherland I. (2001). The Biofilm Matrix - an Immobilized but Dynamic Microbial Environment. Trends Microbiol. 9 (5), 222–227. 10.1016/s0966-842x(01)02012-1 11336839

[B113] Tan L. Li J. Liu X. Cui Z. Yang X. Zhu S. (2018). Rapid Biofilm Eradication on Bone Implants Using Red Phosphorus and Near-Infrared Light. Adv. Mater. 30 (31), 1801808. 10.1002/adma.201801808 29923229

[B114] Taylor P. R. Martinez-Pomares L. Stacey M. Lin H.-H. Brown G. D. Gordon S. (2005). Macrophage Receptors and Immune Recognition. Annu. Rev. Immunol. 23, 901–944. 10.1146/annurev.immunol.23.021704.115816 15771589

[B115] Teitzel G. M. Parsek M. R. (2003). Heavy Metal Resistance of Biofilm and Planktonic *Pseudomonas aeruginosa* . Appl. Environ. Microbiol. 69 (4), 2313–2320. 10.1128/aem.69.4.2313-2320.2003 12676715 PMC154819

[B116] Toyofuku M. Roschitzki B. Riedel K. Eberl L. (2012). Identification of Proteins Associated with thePseudomonas aeruginosaBiofilm Extracellular Matrix. J. Proteome Res. 11 (10), 4906–4915. 10.1021/pr300395j 22909304

[B117] Tran T.-H. Rastogi R. Shelke J. Amiji M. M. (2015). Modulation of Macrophage Functional Polarity towards Anti-inflammatory Phenotype with Plasmid DNA Delivery in CD44 Targeting Hyaluronic Acid Nanoparticles. Sci. Rep. 5, 16632. 10.1038/srep16632 26577684 PMC4649614

[B118] Tzeng A. Tzeng T. H. Vasdev S. Korth K. Healey T. Parvizi J. (2015). Treating Periprosthetic Joint Infections as Biofilms: Key Diagnosis and Management Strategies. Diagn. Microbiol. Infect. Dis. 81 (3), 192–200. 10.1016/j.diagmicrobio.2014.08.018 25586931

[B119] Wagner C. Kondella K. Bernschneider T. Heppert V. Wentzensen A. H??nsch G. M. (2003). Post-traumatic Osteomyelitis: Analysis of Inflammatory Cells Recruited into the Site of Infection. Shock 20 (6), 503–510. 10.1097/01.shk.0000093542.78705.e3 14625473

[B120] Walsh C. (2000). Molecular Mechanisms that Confer Antibacterial Drug Resistance. Nature 406 (6797), 775–781. 10.1038/35021219 10963607

[B121] Wang L. Hu C. Shao L. (2017). The Antimicrobial Activity of Nanoparticles: Present Situation and Prospects for the Future. Ijn Vol. 12, 1227–1249. 10.2147/ijn.S121956 PMC5317269 28243086

[B122] Watnick P. I. Kolter R. (1999). Steps in the Development of a *Vibrio cholerae* El Tor Biofilm. Mol. Microbiol. 34 (3), 586–595. 10.1046/j.1365-2958.1999.01624.x 10564499 PMC2860543

[B123] Weir E. Lawlor A. Whelan A. Regan F. (2008). The Use of Nanoparticles in Anti-microbial Materials and Their Characterization. Analyst 133 (7), 835–845. 10.1039/b715532h 18575632

[B124] Xiang J. Tong X. Shi F. Yan Q. Yu B. Zhao Y. (2018). Near-infrared Light-Triggered Drug Release from UV-Responsive Diblock Copolymer-Coated Upconversion Nanoparticles with High Monodispersity. J. Mater. Chem. B 6 (21), 3531–3540. 10.1039/c8tb00651b 32254448

[B125] Xie X. Sun T. Xue J. Miao Z. Yan X. Fang W. (2020a). Ag Nanoparticles Cluster with pH‐Triggered Reassembly in Targeting Antimicrobial Applications. Adv. Funct. Mater. 30 (17), 2000511. 10.1002/adfm.202000511

[B126] Xie Y. Zheng W. Jiang X. (2020b). Near-Infrared Light-Activated Phototherapy by Gold Nanoclusters for Dispersing Biofilms. ACS Appl. Mater. Inter. 12 (8), 9041–9049. 10.1021/acsami.9b21777 32011117

[B127] Yang H. Y. Li Y. Lee D. S. (2018). Multifunctional and Stimuli-Responsive Magnetic Nanoparticle-Based Delivery Systems for Biomedical Applications. Adv. Therap. 1 (2), 1800011. 10.1002/adtp.201800011

[B128] Yen H. J. Hsu S. H. Tsai C. L. (2010). Cytotoxicity and Immunological Response of Gold and Silver Nanoparticles of Different Sizes. Small 5 (13), 1553–1561. 10.1002/smll.200900126 19326357

[B129] Yoshida S. Hiyoshi K. Oshio S. Takano H. Takeda K. Ichinose T. (2010). Effects of Fetal Exposure to Carbon Nanoparticles on Reproductive Function in Male Offspring. Fertil. Sterility 93 (5), 1695–1699. 10.1016/j.fertnstert.2009.03.094 19446808

[B130] Zanganeh S. Hutter G. Spitler R. Lenkov O. Mahmoudi M. Shaw A. (2016). Iron Oxide Nanoparticles Inhibit Tumour Growth by Inducing Pro-inflammatory Macrophage Polarization in Tumour Tissues. Nat. Nanotech 11 (11), 986–994. 10.1038/nnano.2016.168 PMC5198777 27668795

[B131] Zhang C. Du C. Liao J.-Y. Gu Y. Gong Y. Pei J. (2019). Synthesis of Magnetite Hybrid Nanocomplexes to Eliminate Bacteria and Enhance Biofilm Disruption. Biomater. Sci. 7 (7), 2833–2840. 10.1039/c9bm00057g 31066733

[B132] Zhang X. Bishop P. L. Kupferle M. J. (1998). Measurement of Polysaccharides and Proteins in Biofilm Extracellular Polymers. Water Sci. Technology 37 (4-5), 345–348. 10.2166/wst.1998.0661

[B133] Zhang X. Zhang G. Chai M. Yao X. Chen W. Chu P. K. (2021). Synergistic Antibacterial Activity of Physical-Chemical Multi-Mechanism by TiO2 Nanorod Arrays for Safe Biofilm Eradication on Implant. Bioactive Mater. 6 (1), 12–25. 10.1016/j.bioactmat.2020.07.017 PMC7417618 32817910

[B134] Zoubos A. B. Galanakos S. P. Soucacos P. N. (2012). Orthopaedic and Biofilm. What We Know? a Review. Med. Sci. Monit. 18 (6), Ra89–RA96. 10.12659/msm.882893 22648264 PMC3560733

